# Perspectives of Children and Adolescents on Engaging With a Web-Based Mental Health Program: Focus Group Study

**DOI:** 10.2196/48910

**Published:** 2024-10-15

**Authors:** Christopher Cahill, Jennifer Connolly, Shelley Appleton, Melanie Jade White

**Affiliations:** 1 School of Psychology and Counselling, Faculty of Health, Queensland University of Technology, 149 Victoria Park Rd, Kelvin Grove, 4059, Australia, 61 7 3138 8970

**Keywords:** motivation, demotivation, external motivation, internal motivation, digital health interventions, engagement, internet interventions, mental health, eHealth, youth, children, adolescents

## Abstract

**Background:**

Despite accessibility and clinical benefits, open access trials of self-guided digital health interventions (DHIs) for young people have been plagued by high drop-out rates, with some DHIs recording completion rates of less than 3%.

**Objective:**

The aim of this study was to explore how young people motivate themselves to complete an unpleasant task and to explore perceived motivators and demotivators for engaging with a DHI.

**Methods:**

In this qualitative research study, 30 children and adolescents aged between 7 and 17 years were recruited to participate in 7 focus groups conducted over a 3-month period. Focus group activities and discussions explored sources of motivation to complete tasks and engage in a hypothetical 6-week DHI for anxiety.

**Results:**

Children (aged 7-11 years) reported greater reliance on external motivators such as following parent instruction to complete unpleasant tasks, while adolescents (aged 12-17 years) reported greater internal motivation such as self-discipline. Program factors, such as engaging content, were the most commonly mentioned motivators for engaging with a DHI across both age groups. After that, internal sources of motivation were most commonly mentioned, such as perceived future benefits. External factors were the most commonly mentioned demotivators across all ages, with time commitment being the most frequently mentioned.

**Conclusions:**

The study’s findings have implications for enhancing adherence in future DHIs targeted to children and adolescents. Recommendations include the need for supportive parental involvement for children, while adolescents would likely benefit from mechanisms that promote autonomy, establish a supportive environment, and align with personal interests and values. Belief that a DHI will provide short-term benefits is important to both children and adolescents, as well as having confidence that future benefits will be realized.

## Introduction

Self-guided digital health interventions (DHIs) represent a potential pathway to improve the mental well-being of children and adolescents [1,2]. DHIs use technology to deliver health care and support well-being. DHIs can be fully self-guided (eg, apps and web-based programs) or clinician supported (eg, helplines or web chat) and fulfill a range of purposes such as psychoeducation, cognitive behavioral therapy, self-assessment, or monitoring [3]. Compared to traditional face-to-face therapy, DHIs are inexpensive and widely available; approximately 86% of young Australians have access to DHIs delivered through internet services [4]. However, despite their accessibility and therapeutic appeal, open access trials of DHIs for children and adolescents have been plagued by high drop-out rates, with some DHIs recording completion rates of less than 3% [5].

Posttrial evaluations of DHIs have identified a range of factors that may explain why children and adolescents drop out and fail to complete prescribed web-based treatments. Common reasons include perceived low quality of content, usability issues, lack of support, and privacy concerns [2,6,7]. Although these studies provide useful insights, they tend to focus on program factors such as design, usability, and content, rather than individual factors such as motivation for engagement more broadly [8,9].

From a theoretical perspective, self-determination theory [10] suggests motivation is driven by an interplay of internal and external sources. Applied to motivation to complete DHIs, internal motivators may include engaging in a web-based program because it is enjoyable (eg, “I enjoy the activity”), while external motivators cover a range of motives outside the self, which may or may not interact with internal sources. These include participating in the program to avoid punishment (eg, “If I don’t do it I will get in trouble”), to receive a reward (eg, “I’m doing this to earn a certificate”), or because the program aligns with a partially or fully integrated value (eg, “This program is important to me”) [11]. Previous research has found that higher internal motivation, expressed as treatment readiness, predicted DHI adherence in adults treated for alcohol misuse [12] and completion of a 12-week DHI for anxiety [13]. However, to our knowledge, no previous study has explored how external and internal factors may influence children’s and adolescents’ motivation to complete a DHI. Factors that contribute to “loss of motivation” have also received limited research attention. Described as the “negative counterparts of motives” [14], demotivators represent influences that subtract from an ongoing action [15]. The aim of this study was to explore factors that motivate and demotivate children and adolescents to complete tasks. This knowledge could inform future strategies to enhance motivation for children and adolescents to engage in and complete DHIs.

## Methods

### Study Design

This qualitative study utilized focus group methodology to explore factors that motivate and demotivate young people to complete tasks. This was examined in two contexts: (1) while completing a task in their general life that they find unpleasant or boring, and (2) during hypothetical engagement in a self-guided DHI for anxiety. A focus group methodology was chosen due to the exploratory nature of the research questions.

### Recruitment

Children and adolescents aged between 7 and 17 years were recruited to participate in focus group sessions. The potential for developmental differences in comprehension and expression across the age spectrum [15] was addressed by separating participants into age-based groupings that spanned a maximum of 3 years. Participants were recruited by posting advertisements and news posts on Facebook targeting users residing within 15 kilometers of the university precinct. Study information was also sent to faculty staff throughout the university. Communication material was directed at parents and included both parent and child study information.

### Measures

#### Demographic Questions

Demographic data were collected prior to participation in focus groups. Adolescents (aged 12 years and older) and guardians (for children under 12 years) completed a brief questionnaire containing items on age, gender, year level at school, and history of mental health problems (“Has your child/have you ever experienced an emotional health problem?”).

#### Qualitative Questions

To support expression and mitigate the risk of groupthink [16], participants were encouraged to draw or write their ideas in response to stimulus material presented by the moderator (Textbox 1).

Textbox 1. **Question guide.**

Part A: Motivating Self

Think about something that you have to do each week that you really don’t like doing, but have to do anyway. Something you have to get done by yourself without help from anyone else.

1. Draw yourself doing it.
2. Put some feeling stickers on there.
3. Draw or write what it is that makes you get it done.

Part B: Engaging with Digital Health Interventions

I want you to imagine that you have signed up for this program, because you’ve been feeling scared, worried, or sad. To do the program, you have to log in on your computer every week for 6 weeks.

1. Start by putting some feeling stickers on there.
2. What do you think about having to keep going with it? Are there any reasons you don’t want to keep doing the program each week? What are the thoughts going through your head about that?
3. What are some things in your life that might get in the way of you logging on and doing the program?
4. What are some reasons why you would keep doing the program every week, even if you didn’t feel like doing it?
5. Draw or write down some things that might help you keep going with the program, even if you don’t feel like doing it?

### Procedure

Eligible participants self-referred to the study by completing a web-based registration form. They were then contacted by email by a member of the research team and offered a place in a focus group based on availability specified during registration. Focus groups were held at a Brisbane, Australia, university campus between October and December 2021. On arrival, written consent was obtained from parents or guardians of participants under 16 years while those aged 16 and 17 years provided their own written consent. The demographic survey was completed by parents or guardians of children younger than 12 years, while adolescents aged 12 years and older completed their own. Participants were seated evenly around a large table, accompanied by a moderator (CC) who steered the discussions, as well as a supporting investigator (JC or SC, both clinical psychologists) responsible for note-taking and offering assistance to any child requiring attention during the focus group session. Participants were grouped together with children and adolescents of comparable ages to facilitate a conducive environment for interaction and sharing.

**Figure 1.**
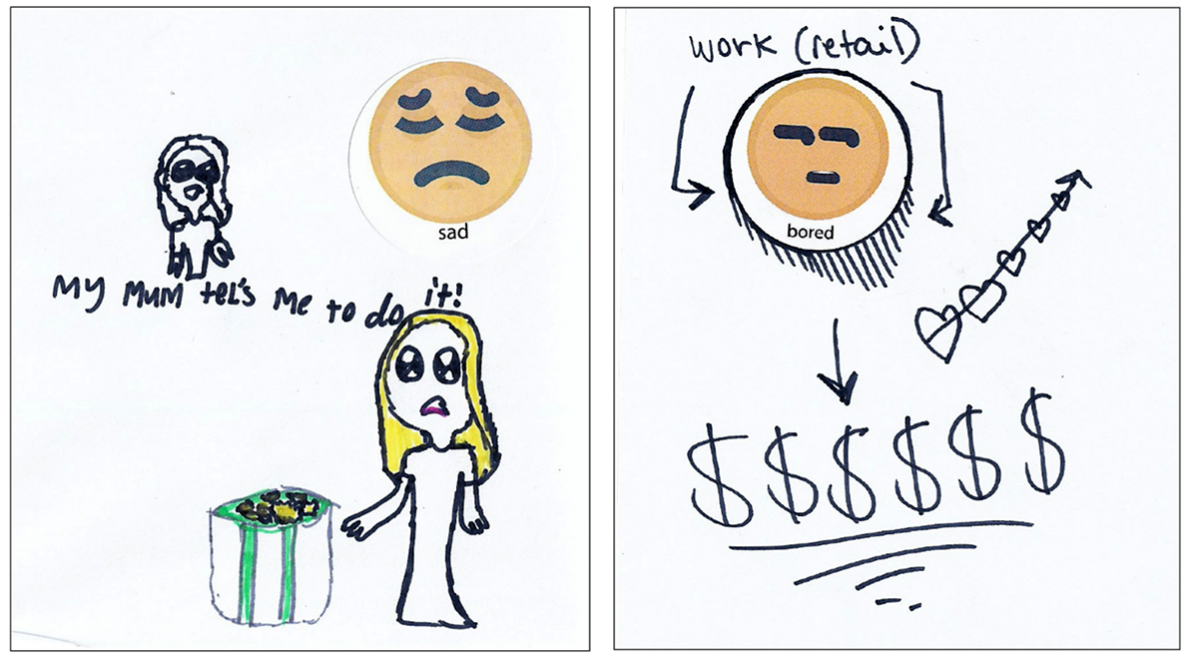
Examples of participant drawings about motivating self. (A) The left drawing was created by a 7-year-old female participant. (B) The right drawing was created by a 15-year-old female participant.

Parents were invited to remain in the room at a distance from the group or return at the conclusion of the session. Each focus group included two creative activities. In the first task, participants were provided with a blank piece of A4 paper, a set of 12 feeling/emotion stickers, and a pack of colored pens. They were invited to draw or write about a regular task they dislike doing and to then indicate how they felt about doing it using the stickers provided or by drawing their own. They were then asked to draw or write what it is that makes them get the task done. After completing their sheet, participants were invited to share their picture with the group if they were comfortable doing so (see Figure 1 for sample outputs).

**Figure 2.**
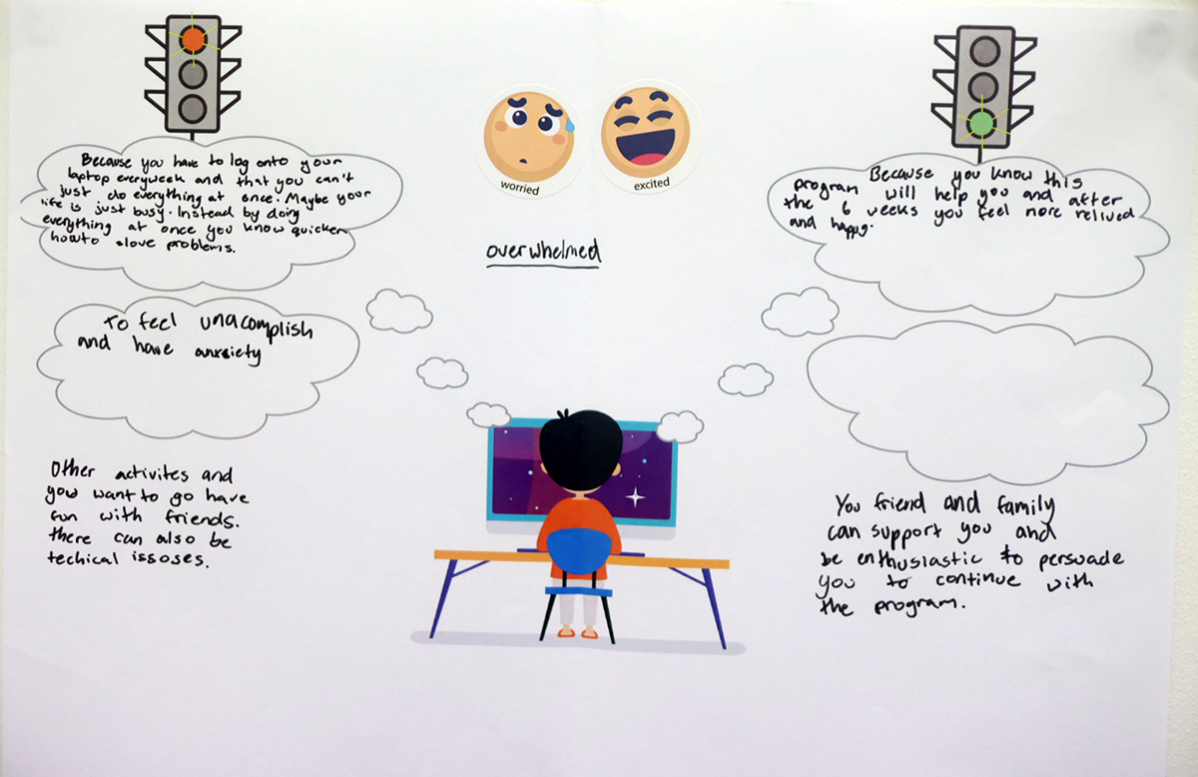
Example of a participant’s drawings about engaging with a digital health intervention, created by a 12-year-old female participant.

For the second task, participants were provided with an A3 piece of paper featuring a color graphic of a young person facing a computer screen with thought bubbles to the side, a second set of 12 feeling/emotion stickers, and a pack of colored pens. Participants were introduced to a hypothetical DHI and shown a sample image of the dashboard of a web-based program for youth anxiety currently under development. We purposefully maintained a level of ambiguity regarding the application to prevent any potential biasing of participants’ responses. Participants were asked to imagine they had signed up for the program because they had been feeling anxious. Participants completed 5 activities to draw out internal and external motivators and demotivators. They first indicated how they felt about doing the program using the sheet of stickers. They were then asked to write in one of the thought bubbles what they thought about doing it and any reasons why they would not want to continue with the program. Following this, they wrote or drew any things that would get in their way. Next, they were asked to use another thought bubble on the other side of the page to write down any reasons why they would keep doing the program even if they did not want to do it. Finally, they were asked to write or draw some things that would help them keep going with the program and get it done (see Figure 2 for an example output). The procedure for sharing and discussion was similar to the first task. All focus group sessions were audio-recorded and lasted between 60 and 80 minutes (including a break). A pilot test was conducted with 3 children to test engagement with drawing activities and general task comprehension. No changes to the guide were considered necessary; pilot group responses were not included in the analysis.

### Data Analysis

Audio recordings from each focus group were transcribed and entered into QSR NVivo computer software (version 13; Lumivero). Thematic analysis [17] was used to analyze the data. Research team member CC initially reviewed and coded the transcripts. These codes were reviewed by a second researcher (SA) and both researchers independently generated preliminary themes. Any theme discordance was reconciled in discussions with a third researcher (JC). The final step involved all authors reviewing the theme labels and quotes for consistency and relevance and grouping subthemes.

### Ethical Considerations

The study was approved by the Queensland University of Technology University Human Research Ethics Committee (Reference 4312-HE31). Written consent was provided by guardians of children under 16 years while those aged 16 and 17 years provided their own written consent. Participant responses were deidentified prior to analysis. Participants and their guardian were compensated with gift cards valued at Aus $30 (US $20.30) and Aus $40 (US $27.07), respectively.

## Results

### Participant Characteristics

A total of 31 participants were recruited; however, one (female, 16 years) was excluded due to inability to participate in the focus group due to severe anxiety. The final sample consisted of 30 children (18/30, 60% female) in 7 focus groups, with group sizes ranging from 3 to 6 participants (mean 4.3, SD 0.96). The age range within each group spanned 1-3 years. Specifically, the groups were conducted with participants aged 7-9 years, 7-10 years, 8-10 years, 10-13 years, 11-14 years, 14-16 years, and 16-17 years. Demographic characteristics are presented in Table 1.

**Table 1.** Participant characteristics.

	Children (7-11 years)	Adolescents (12-17 years)	Total (N=30)
Participants, n (%)	17 (57)	13 (43)	30 (100)
Female, n (%)	11 (65)	7 (54)	18 (60)
History of emotional problems, n (%)	8 (47)	7 (54)	15 (50)

### Motivation to Complete Unpleasant Tasks

Home-based chores and routines (eg, unloading the dishwasher, taking a bath) were the most common unpleasant task type identified by children (12/17, 70%). In contrast, the majority of adolescents (9/13, 70%) chose tasks that originated out of the home (eg, homework or a part-time job).

### Emotions

Participants selected an average of two stickers to represent their feelings about doing the task (mean 1.8 stickers, SD 0.8). Feeling bored (16/30, 53% of participants) and annoyed (15/30, 50%) were the most commonly selected feelings associated with things they did not like doing but had to do anyway. The popularity of these two emotions was common to both children and adolescents.

### Motivational Sources

As shown in Table 2, children and adolescents identified a range of internal and external sources that underlie their motivation to complete unpleasant tasks. Adolescents were generally more expressive than children, identifying an average of 2.8 (SD 1.2) versus 1.1 (SD 0.9) sources, respectively. Internal motivation sources outnumbered external sources for adolescents, while external sources were more frequently mentioned by children.

**Table 2.** Internal and external motives experienced by children and adolescents during an unpleasant task.

	Number of responses from children (n=15)	Number of responses from adolescents (n=13)	Total (n=28)^a^
**Internal motivational sources**	4	28	32
Self-discipline	2	14	16
Sense of achievement	1	6	7
Fear of negative consequences	0	6	6
Making it fun	1	2	3
**External motivational sources**	12	9	21
Receive a reward	7	2	9
Avoid parental disapproval	1	4	5
Follow parent instruction	4	0	4
Social desirability	0	3	3
**Total**	16	37	53

a The data represent participant responses. Two children did not share motivational sources.

### Internal Motivational Sources

The most common internal motivation source across all focus groups was self-discipline (16 responses). Included in this theme were reflections on responsibility and strategies that young people use to stay on task, including the use of self-talk and choosing to focus on the moment, as shown in the following comments.

*I’ll be like, “Come on man, you’ve committed to these subjects, You need to do it.” And so I feel like that’s a bit of my discipline*.

[4R-17]

*Yeah, you kind of just have to, so you force yourself to.*

[6J-15]

*Just kind of thinking, like, “This is my responsibility and it’s helping me by doing it.*

[5G-13]

A sense of achievement derived from completing the task was another common theme, expressed in comments from 6 adolescents and 1 child in the focus group discussions.

*But when I have managed to finish it, then I’ve actually spent my time doing something useful, like I’ve achieved something*.

[3Z-13]

*It feels rewarding, so then sometimes I use a bit of that, like I tell myself, oh come on, just get it done and then you’ll feel better afterwards that you’ve just done it*.

[4R-17]

In addition, 6 adolescents (46% of adolescent participants) stated they were motivated by considering the negative consequences of not completing the task. For several participants, this appeared to be linked to a previous unpleasant experience (their own or observed in others). There was one adolescent that mentioned fear of failure and demonstrated the use of positive reappraisal as their language changed from fear of failure to focusing on the positive in order to motivate themselves to complete a difficult task.

*So basically, I just remind myself of examples of people around me and just kinda kick myself up the pants.*

[6L-16]

*I’m just like, “If I don’t do this, then it’ll lead to this.” So, it’s just kind of, “Do it so that stuff doesn’t happen.*

[6E-14]

*And then fear, I don’t wanna get a bad end result, year 12. I need to put in a lot of effort into these things if I wanna do well…*

[4R-17]

There were 3 participants who mentioned using various methods to make the task fun, such as thinking about other things or using music, either by listening to it or singing.

One thing that just pushes me to make sure I do it is I just think about other things while I do it.

[3E-13]

We make up a little song and then it helps us like, and then we like, sing it and then we get it done really quickly.

[7A-9]

### External Motivational Sources

The most frequent external motivation was to receive a reward. For children, this often represented the opportunity to do other things (7 responses), while adolescents referred to financial rewards for unpleasant jobs (2 responses).

*After I do it I can play.*

[2E-10]

*And something that helps me get through it is that I realize what I could do after I’m done, like, something good I can do it after I’m done.*

[5S-11]

*Obviously motivated by money ‘cause yeah, that’s the only reason people work.*

[6J-15]

Parent or guardian influence on a child’s motivational decision-making was evident in several responses, including inclination to follow parental instructions (4 responses).

*Well, I normally ask my mom: “What do I have to do?”, I do it.*

[7E-9]

*My mom told me to, so I have to do it.*

[1H-10]

Avoiding parental disapproval from parents or guardians was mentioned by 4 adolescents and 1 child.

*I sometimes don’t have time to do it, which makes like my dad or my mom mad because I haven’t done my chore.*

[3E-13]

*…and the thing that motivates me to do it is my parents will get mad at me if I don’t do it.*

[3L-14]

*I don’t get in trouble, so I have to do it.*

[1J-8]

Social desirability was mentioned by 3 adolescents, 2 of whom highlighted peer comparison, while the third was motivated by a desire to meet the expectations of their parents and people around them.

*I’m quite competitive and I know if I’ll get a bad mark or compared to other people I’ll get pretty sad so I just make myself do what I can.*

[6E-14]

*I’ll say with me, my friend group is also pretty academic, so you think, “Oh, if I don’t get a good mark, then other people are gonna be, “Oh, why are they friends with her? She’s not as smart as them.” You see.*

[6V-15]

Of course, (I’m concerned about) my parents and then the people around me.

[6E-14]

### Engaging With a DHI

**Figure 3.**
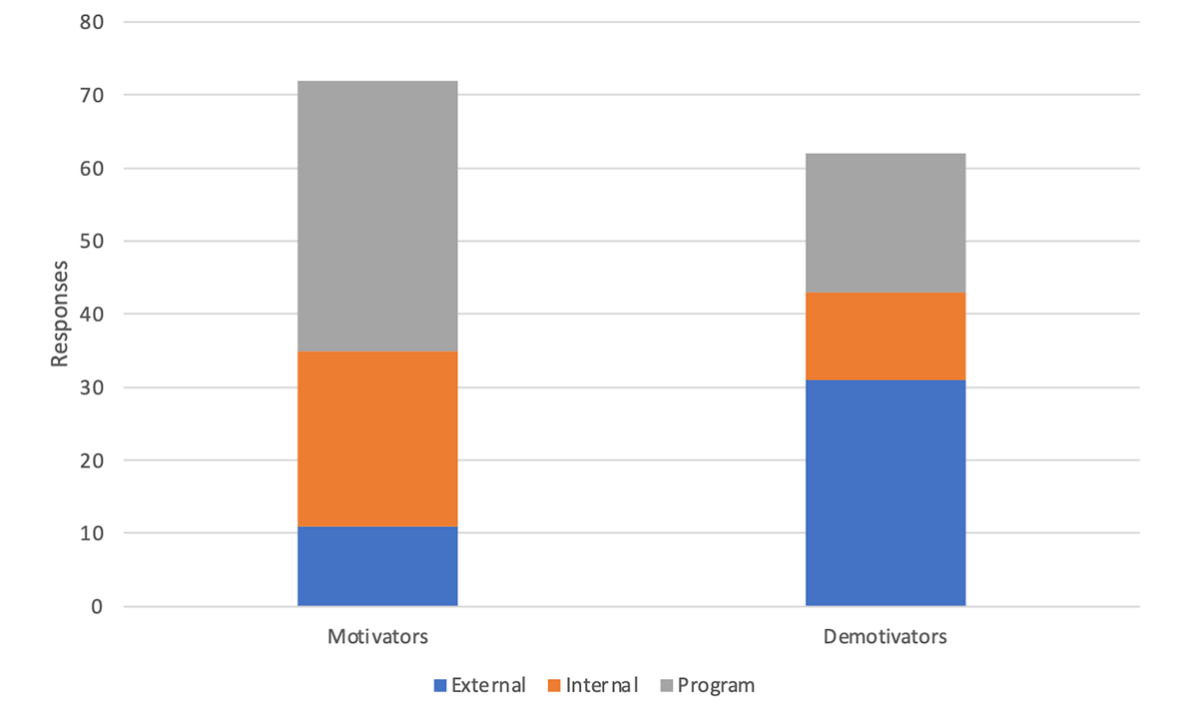
Motivation sources to engage in digital health interventions.

Participants identified a range of motivating (72 responses) and demotivating (62 responses) sources that were categorized into internal, external, and program factors. As shown in Figure 3, program factors were the most frequently mentioned motivators (34 responses), while external sources were the most frequently mentioned demotivators (31 responses).

### Emotions

Participants selected an average of 3 emotions to represent how they would feel about completing a 6-week DHI (mean 3.1, SD 1.3). The 5 most common emotions reflected a mix of negative emotions (15/30, 50% were worried; 10/30, 33% anxious; 9/30, 30% scared) and positive emotions (13/30, 43% excited; 12/30, 40% happy).

### Motivating Sources That Support Engagement in DHIs

As shown in Table 3, participants identified a range of motivational sources they perceived would help them complete a 6-week DHI. The highest number of responses were program-specific factors (37 responses), followed by internal sources (24 responses) and external motivational sources (11 responses). Across all categories, perceived future benefits (14 responses), engaging content (10 responses), and gamification (7 responses) were the most commonly mentioned motivators.

**Table 3.** Sources of motivation to engage with digital health interventions.^a^

	Number of responses from children (n=17)	Number of responses from adolescents (n=13)	Total (N=30)
**Internal**	7	17	24
Perceived future benefits	5	9	14
Sense of accomplishment	1	4	5
Commitment	1	4	5
**External**	4	7	11
Social pressure	0	4	4
Social support	1	3	4
Rewards	3	0	3
**Program factors**	11	26	37
Gamification	0	7	7
Program reminders	2	3	5
Rewards in the program	0	6	6
Incremental benefit from each component	3	2	5
Engaging content	5	5	10
Program design features	1	3	4
**Total**	22	50	72

a The data represent participant responses.

### Internal Motivators

Perceived future benefits of the program were the most commonly cited motivating factor. This theme covered several related concepts, including expectations of improved mental health, a sense of confidence they will be happier in the long run, improved coping self-efficacy, and a desire for self-improvement. Although most responses were from adolescents (9/14, 64%), participants as young as 9 years old expressed a relatively long-term view. Notably, child responses expressed confidence (denoted by the use of words such as “you know”), while adolescents appeared to temper belief (“there is a chance it could work”).

And you know you’ll be much happier after and you feel loved and safe.

[1A-10]

I might not like this, (but) it will help me in the long run.

[7A-9]

The desire of better mental health, that’s self-motivated. I wanna get that kind of thing self-improvement.

[4E-17]

Probably the chance it could actually work. So, obviously after the first one, if I feel like maybe there is a chance it will work out, I’ll be more committed to keep doing it.

[4R-17]

Among all participants, 5 suggested that thinking about the DHI as an accomplishment would help motivate and inspire them to complete the program.

Accomplished feelings, I guess, like your mental health is kind of improved, and if it feels kind of easy to keep going, then it makes it a whole bunch easier to keep going.

[5G-13]

I would be proud of myself for actually sticking to the program. It would make my parents happier, and I would have achieved something.

[3Z-13]

Classified under the theme of commitment, 4 adolescents and 1 child stated they felt obligated to finish what they started.

And then like think just guilt of giving up. I feel like I would just let myself down and then probably, just my…. Just discipline…. Oh, I made a commitment, I need to do it.

[4R-17]

Since it’s like a program. I think I’d kinda feel obligated to finish it so afterwards

[6V-15]

Just like five weeks to go or something, like…

[3C-11]

### External Motivators

External influence from others, expressed as social support (eg, encouragement from family) or social pressure (eg, pressure to meet expectations from parents), represented the most commonly mentioned external motivator by participants. A total of 4 participants identified family members or therapists as a source of social support to complete a DHI, as highlighted in the following responses.

Friends and family that can help support you and keep you…keep enthusiasm up.

[5A-12]

Having your parents motivate you.

[3L-14]

There were 4 adolescents who identified aspects of social pressure from parents and therapists would influence them to complete the DHI.

Parents making me, therapists making me. If they’re like, “You have to,” then I’m like, “Okay, I have to.

[6J-15]

And then there’s the external pressure, I guess it’s like no external pressure, but wanting to make others happy.

[4E-17]

Rewards for participation, including games and tangible treats, were identified by 3 children as potential sources of motivation to complete the DHI.

*Like getting a reward afterward. Like getting to play games or something*.

[2E-10]

*Food.*

[3C-11]

### Program-Specific Motivators

Engaging content was the most common program-specific motivator mentioned by participants. Participants believed that a DHI program that was fun (3 responses), enjoyable to use (3 responses), and interesting (2 responses) would encourage them to complete the assigned program. Almost one-third (5/17, 29%) of child participants made a comment about engagement, making this the most common facilitator within the younger age group.

You could have enjoyed last time, so you can’t wait to come back.

[1A-10]

If you see something exciting you will have a go at it. It might be really exciting, so you’ll just keep doing it.

[2V-8]

A program that offered tangible rewards for completion was recognized by adolescents (6 responses) as something that would help them complete a multisession DHI over 6 weeks.

Well, maybe if it was in the actual thing. Each one you get completed, that could be like, not necessarily a real-life reward but something in the game.

[6E-14]

Probably if I felt like there was a reward. If I wasn’t doing it just for my own personal gain. If I felt like there was actually some kind of tangible thing at the end that I get.

[4R-17]

A voucher. If you finish a full six weeks.

[6J-14]

Gamification elements (eg, integrated games, end goals, and customizable avatars) were identified by 7 adolescents as program features that could support engagement. This theme was not mentioned by children.

Just more games to engage in, then maybe a couple more things during the week. Just so it can keep your mind off the mental illness

[6J-14]

People like avatars, customizing avatars. That was a thing when I was a kid.

[6J-15]

A total of 5 participants mentioned that they would persist with a DHI that demonstrated incremental benefits from what is learned in each component. Although one adolescent explicitly described the benefit as a “feeling of progress,” other participants explained they would be willing to continue if the program was teaching them something or providing strategies they could immediately use to improve their mental health.

It might be helpful, so it can teach me something.

[1J-8]

I will have…. After doing the first week of the program, if I find it fun to do and I find the topics interesting and I’m learning about it, I’d be willing to continue doing it and have fun at it.

[4A-16]

And you know that it helps relieve stress, and they give strategies to manage your anxiety.

[5S-11]

Feeling of like progress. Like I’m actually getting somewhere.

[4R-17]

There were 4 participants who identified that program design features, including easy access and short session duration (one participant suggested a 15-minute time frame), may increase engagement in a DHI.

Just easy to access on your computer and stuff.

[5G-13]

I don’t really know too much about it but if it’s a small time like 15 minutes at a time or whatever then I’m like, “Okay, well, I might as well.”

[6J-15]

Stuff like maybe an easy URL to type up or something.

[5G-13]

A DHI program that reminded users to log in was identified as a potential motivator by several adolescents and 1 child.

Something that might help me keep on doing it is gentle reminders, to do it throughout the week, or encouraging messages.

[3Z-13]

The things that can motivate you, like a word on motivation, reminders, like it being fun to people, encouraging phrases.

[3C-11]

### Demotivating Sources

As shown in Table 4, time commitment (25 responses) was the most commonly mentioned demotivator. Other demotivators typically mentioned were apathy toward continuing (7 responses), concerns about effectiveness (7 responses), and content engagement (6 responses).

**Table 4.** Sources of demotivation to engage with digital health interventions.^a^

	Number of responses from children (n=17)	Number of responses from adolescents (n=13)	Total (N=30)
**Internal**	5	7	12
Anxiety	1	1	2
Negative emotions	2	1	3
Apathy toward continuing	2	5	7
**External**	12	19	31
Access to equipment	0	2	2
Lack of social support	0	4	4
Time commitment	12	13	25
**Program factors**	4	15	19
Concerns about effectiveness	1	6	7
Content engagement	1	5	6
Length of program	0	2	2
Privacy concerns	2	2	4
**Total**	21	41	62

a Data represents a count of responses, not participants.

### Internal Demotivators

Apathy toward continuing was identified in focus groups with both adolescents (5 responses) and children (2 responses). Children described apathy in terms of “can’t be bothered” or “you don’t want to do it,” while adolescents used the term “motivation” and reflected on a sense of disregard for the program.

And then just again…. Just my own motivation, how much I’d care.

[4R-17]

And then just generally your own self-motivation to do it.

[6J-14]

Several adolescents linked lack of motivation to depressive symptoms, as shown in the following comments.

General mental illness, a lack of motivation, if I’m physically not able to do it, I’m not up to it, then I’m mostly not going to.

[6J-15]

Or maybe just your feelings or thoughts, ‘cause if you have depression in the first place you might not be very happy.

[3L-14]

Comments from 3 participants indicated that negative emotions connected to needing help could act as potential barriers to completing a DHI. These feelings included shame and hopelessness.

On the red side, the emotions could be like ashamed at yourself...

[3C-11]

Disappointed at the fact that they need this extra support, if it’s something.... If it’s obvious enough that this is a mental health thing.

[4E-17]

You don’t believe anything will make you feel better.

[1A-10]

Recognizing that the hypothetical DHI would be used by people with anxiety, 2 participants highlighted that participation in the program may exacerbate their anxiety, which could be a barrier to completion.

The kind of thoughts that would stop me would be lots of anxiety about it, like feeling unaccomplished, like I haven’t kind of done anything, and if it feels really hard to keep going.

[5G-13]

And you are worried about sharing your ideas.... Thinking about the past. If something’s happened in the past, something like anxiety.

[5S-11]

### External Demotivators

Concerns about time was the most common reason participants would not engage with the program. Time commitment encompassed a range of related obligations that impacted young people’s perceived available time, including schoolwork, part-time work, social activities, household chores, and fatigue resulting from these tasks, as highlighted in the comments below.

Because you can be really busy, and...because you said how it’s every six weeks, and maybe a person doesn’t have that much of time.

[5A-12]

It takes time away from me. It might not even help me, and it’s a waste of energy.

[3Z-13]

I swim. I’m really busy. I do athletics and I’m really tired after school.

[1A-10]

Just school and work and just stuff outside like extracurriculars and stuff.

[6V-15]

Mirroring the motivating factor of social support identified above, lack of social support was identified as a demotivator faced by adolescents (4 responses).

Peer groups, I guess, if they’re not that supportive. That could be peer pressure.

[5G-13]

There’s a lack of support from family, well that’s I think the main thing that would get them doing this in the first place. So, if that support begins to wear off then they are just not gonna keep doing it.

[4E-17]

Concern about access to equipment that was perceived to be necessary to complete the program was identified in 2 comments by adolescents.

And then objective things like access to an electronic device and access to the internet.

[4E-17]

So it’s just a bit more of a hassle if I’m having to do that at my dad’s, I’d be more discouraged to do it.

[4R-17]

### Program-Specific Demotivators

The most common program-specific demotivator was concerns about program effectiveness. Responses from 7 participants suggested that any doubts about perceived effectiveness of the program would undermine their motivation to complete a DHI.

Probably doubt, like “Is this actually gonna work? Is this worth my time?”

[4R-17]

That I’ve had to do this kind of stuff before, and it never really does anything so what’s the point? And then I’m not sure ‘cause obviously I haven’t done it.

[6J-15]

Similar to concerns about effectiveness, young people highlighted the importance of content engagement (6 responses), suggesting that repetitive, uninteresting, unenjoyable, or patronizing content would diminish motivation to complete DHIs.

If it just feels it’s sort of the same thing again every five weeks, I’ll lose motivation to keep doing it. If I feel.... It’s not like...getting anywhere.

[4R-17]

Most likely not keep doing the program. If I was not learning anything I find interesting. So, I’d probably be bored and I wouldn’t wanna come again.

[4A-16]

If it’s too childish then I’ll feel kind of patronised and annoyed so that’s a hypothetical based on what it’s actually like.

[6J-15]

Comments from adolescents highlighted that privacy is crucial to users of DHIs, and anxiety related to potential privacy breaches could be a major demotivator for users.

That’s a massive problem with people who have anxiety and depression, ‘cause they don’t want people to know.

[6J-14]

Yeah they start panicking that it’s gonna get leaked or something, yeah.

[6J-15]

The length of program was identified as a potential demotivator by 2 adolescents who felt that young people with symptoms of anxiety or depression may find it difficult to commit to ongoing, web-based therapeutic sessions.

And why am I spending so much time on it? It’s like five weeks is a long time.

[6E-14]

If you know of anxious children, I’m sure something like being on a computer for maybe like an hour at a time, would be a daunting task.

[4E-17]

## Discussion

### Principal Findings

This study aimed to investigate factors that motivate and demotivate children and adolescents in completing challenging tasks with the goal of informing future strategies to enhance engagement in DHIs. We found that when faced with a task they do not want to do, children (7‐11 years old) tended to be motivated by external sources. Short-term extrinsic rewards were the most common source, evidenced by children’s desire to get the task done so they can do other things. In comparison, internal sources were more commonly reported by adolescents, who commonly spoke about the importance of *s*elf-discipline and a sense of accomplishment.

There are several factors that may explain the difference in motivational source between children and adolescents. First, it is likely that as children age and gain autonomy, they have more opportunities to face challenges, develop coping strategies, and derive internally regulated motivation, while younger children have limited experience beyond seeking support from caregivers [18,19]. Another factor that may explain the differences between age groups was task context, such that most adolescents chose a learning task, which offered potentially greater rewards for effort (ie, attainment value) compared to household chores, which were the common choice for children.

The findings from this study suggest that adolescents draw equally on positive motivations (achievement and reward) and negative motivations (fear of negative consequences and punishment) to persist with everyday unpleasant tasks. Although previous research tends to suggest that positive motivations are associated with greater long-term well-being, the findings from discussions with adolescents show that negative motivations can lead to positive outcomes. This is consistent with several studies that have shown that negative motivation (ie, fear of failure) can support positive change in children and adolescents [20,21].

Turning to the challenge of completing a hypothetical 6-week DHI program, perceived future benefits of the program was the most frequently mentioned motivation theme across both age groups, though the theme was more commonly mentioned among adolescents. This aligns with educational studies that have observed that differentiation between interest in and importance of academic tasks grows with age, as adolescents see beyond immediate desire for engagement (“will I enjoy it?”) and may be more motivated by attainment value (“will this help me?”) [22].

According to expectancy-value theory of achievement [22], persistence requires that young people not only recognize future benefits, but also believe that those outcomes are achievable. In this study, belief in a positive outcome was expressed in child and adolescent focus group discussions. Closely linked to the concept of success expectancy [22], belief is widely recognized as a predictor of adherence in therapeutic settings [23]. Previous research suggests that success expectancy—the belief that one will be successful in achieving a desired outcome—may be amplified by personal interaction [24], a factor that is not offered in self-guided DHIs. This underlines the challenge faced by DHIs to build belief in an online environment where personal contact with therapists or counselors is not available. Although therapist support is exogenous to self-guided DHIs, social support provided by family and friends may inspire belief. Social pressure was also mentioned as an external motivational source among adolescent participants. This is consistent with developmental studies that have shown social influences (positive and negative) are amplified during adolescence, when young people become increasingly exposed to a range of contextual factors, including social comparison in peer relationships, school culture, and family involvement [25]. Previous reviews have investigated the use of social platforms as a DHI program feature (ie, texting, social networking, web-based message boards, discussion forums) to support engagement [2]. However, our findings suggest that young people value support from family members as an important external motivation source rather than a feature embedded in the DHI. Family support strategies have shown to be effective for addiction recovery [26] and eating disorders [27] in adolescents, therefore a similarly designed family-based social support strategy may assist adolescents to complete self-guided DHIs.

Consistent with previous research [2,9], our findings highlighted that engaging content was considered an important motivator for both children and adolescents. For children, engaging content was the most mentioned motivator to stay engaged in a multisession DHI. Although also important, adolescents valued other program features more highly, including gamification and tangible rewards (such as certificates and rewards outside the DHI). However, given the nature of this study, it is not possible to make inferences about the relative importance of content versus gamification, although the observed difference between age groups may be explained by children having less exposure to games-related content online compared to older participants.

Time commitment was the most frequently mentioned demotivator in both child and adolescent groups. This result corresponds with previous DHI reviews where “lack of time” [28], “inability to find time” [9], and “time constraints” [29] were raised as issues by young people. This suggests that the benefits of convenience and accessibility offered by DHIs compared to traditional therapies [30] may not be valued by young users. Outside of time commitment, adolescents also expressed concerns about effectiveness. This is in line with a recent systematic review of DHIs, which identified “credibility” in relation to evidence of the intervention’s effectiveness as a common contributor to high retention rates for children and young adults [2].

### Implications for the Development of Youth DHIs

Our focus group findings led to a number of recommendations for improving adherence in future DHI developments for children and adolescents. For children, engaging content combined with immediate, extrinsic rewards are important motivators to complete a DHI. Children also demonstrated a capacity to seek attainment value in a difficult task, however this needs to be nurtured with developmentally appropriate language as the majority of children did not consider the future benefits of a DHI in our discussions. Another implication for DHI designers is that children seek pleasurable online experiences at each point of engagement, while adolescents may respond more effectively to strategies designed to reinforce attainment value of the therapy goal (such as progression charts). The involvement of parents/guardians also appears to be a motivator valued by children, who identified parental instruction, approval, and reward-seeking in our focus group discussion. This highlights the importance of continuous parental engagement [31] in DHIs for children, which may include coaching-style support and the administration of rewards upon completing each step of a multisession program.

For adolescents, a sense of achievement was an important internal motivator for completing tasks they do not want to do. Recognizing that sense of achievement is closely linked to an adolescent’s sense of personal control [32], future DHIs can benefit from strategies designed to provide autonomy, create a supportive environment, and connect with adolescents’ interests. Granting autonomy can increase engagement with DHI tasks. This can be achieved by allowing them to select topics aligned with their interests. Strategies designed to provide social support from family and friends may help to amplify attainment value and buffer against concerns they may express about program effectiveness. Youth DHIs should also seek to incorporate interests into the content whenever possible, and tailor sessions and homework to align with their extracurricular interests. By making the program personally relevant, adolescents are more likely to see the value in completing the program.

Another implication for DHI designers is that negative motivations are frequently expressed by adolescents. In particular, fear of negative consequences was used for completing tasks they do not want to do. Negative motivations can provide initial drive; however, available evidence suggests that negative motivations may not sustain longer-term engagement [20]. Although acknowledging the importance of consequences for nonadherence, DHI programs need to provide mechanisms for adolescents to address these negative motivations in positive ways while also building self-confidence. Strategies could include providing mechanisms for adolescents to set their own deadlines, inviting others to hold adolescents accountable, or establishing tangible repercussions for inaction.

Finally, DHI developers also need to consider practical strategies to address concerns that both children and adolescents have about time required to complete a DHI. The issue of time commitment could be addressed in several ways, including providing brief, targeted content that can be quickly consumed on a regular basis. DHIs should also consider ways to integrate content with other applications and smart devices (eg, wearables) that children and adolescents access regularly to reduce learning time and support busy lifestyles, in and out of the home.

### Limitations

Several limitations need to be acknowledged. First, while having a history of mental health or emotional problems was common in our sample, they were not a DHI-seeking population. Generalization of results must therefore be approached with caution. Another limitation was that participants were not required to have previous experience with DHIs. Some responses from children reflected limited understanding of DHIs and web-based programs more generally. This implies that differences in responses from older participants may reflect online experience rather than developmental differences. Future work should assess children’s and adolescents’ perspectives in the context of their prior DHI and online experience more generally. Another potential limitation was group structure. Some children chose not to share their story about their artwork and so there was some potential loss of data. Although the moderator actively redirected conversation to younger participants, older participants were the predominant voices in most groups, and this may have contributed to a lack of sharing.

### Conclusions

Results of this study indicate that engagement strategies that engender belief that a DHI will provide short-term benefits are important to both children and adolescents. Additionally, confidence that future benefits will be realized was also articulated by adolescents, but this appeared to be less important to children, reflecting potential age-related responses and online experience. The study also found that social support may be an effective source of engagement for young people; however, more research is required to explore how persuasion from family members can nurture belief and confidence in future benefits from DHIs. Finally, future research should consider how engagement strategies can be used to shift children’s and adolescents’ perception that self-guided DHIs are time-consuming.

## Abbreviations

DHI: digital health intervention
